# Supplementary figures and images for: Transcriptomic correlates of electrophysiological and morphological diversity within and across excitatory and inhibitory neuron classes
Source: PLoS Comput Biol. 2019 Jun 18;15(6):e1007113. doi: 10.1371/journal.pcbi.1007113 (PMC6599125; doi:10.1371/journal.pcbi.1007113)

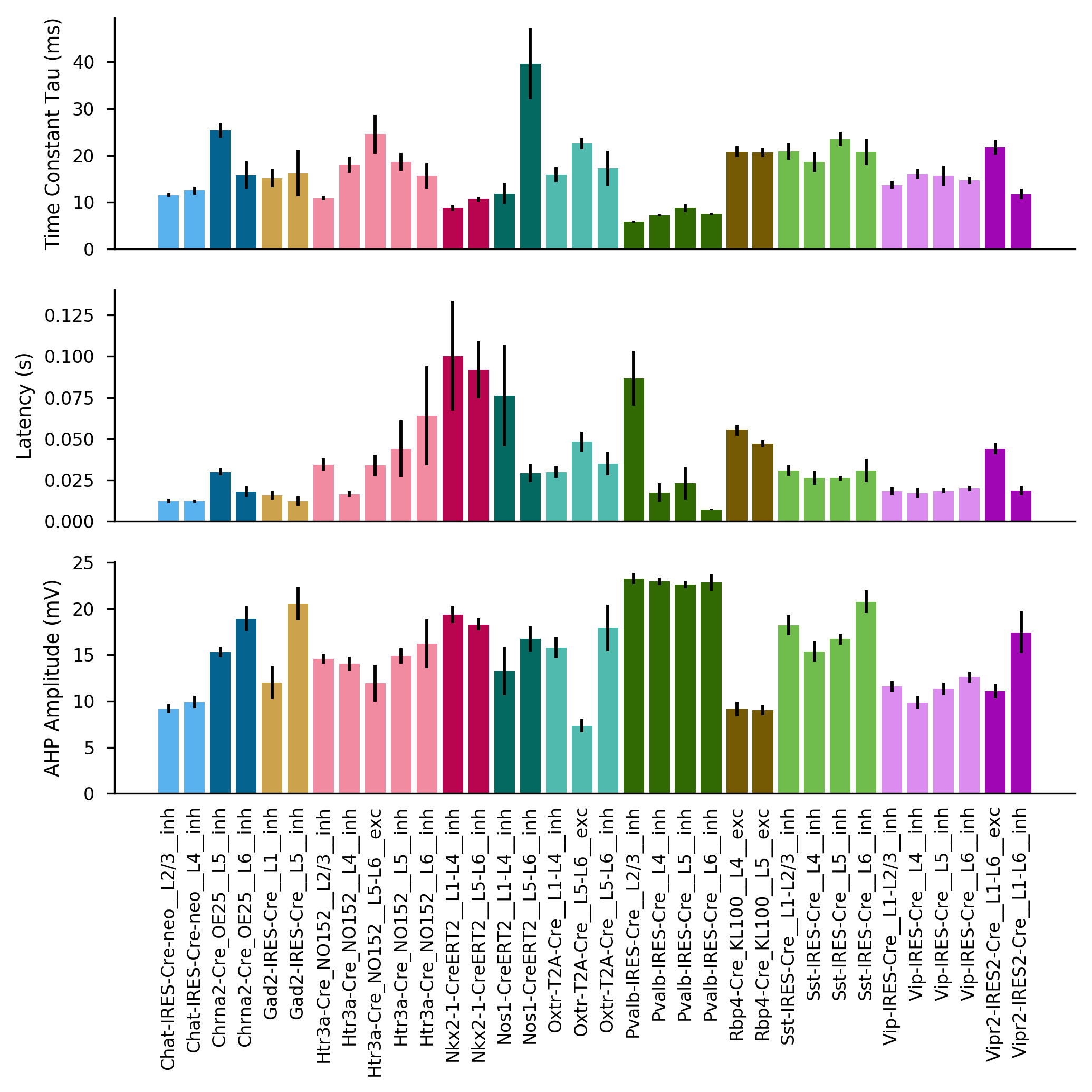

Supplement: S1 Fig — Cell types defined based on the same Cre line but different layers and/or excitatory/inhibitory identity show differences in electrophysiological features. Data are represented as mean ± SEM. (TIFF) [file pcbi.1007113.s001.tiff]

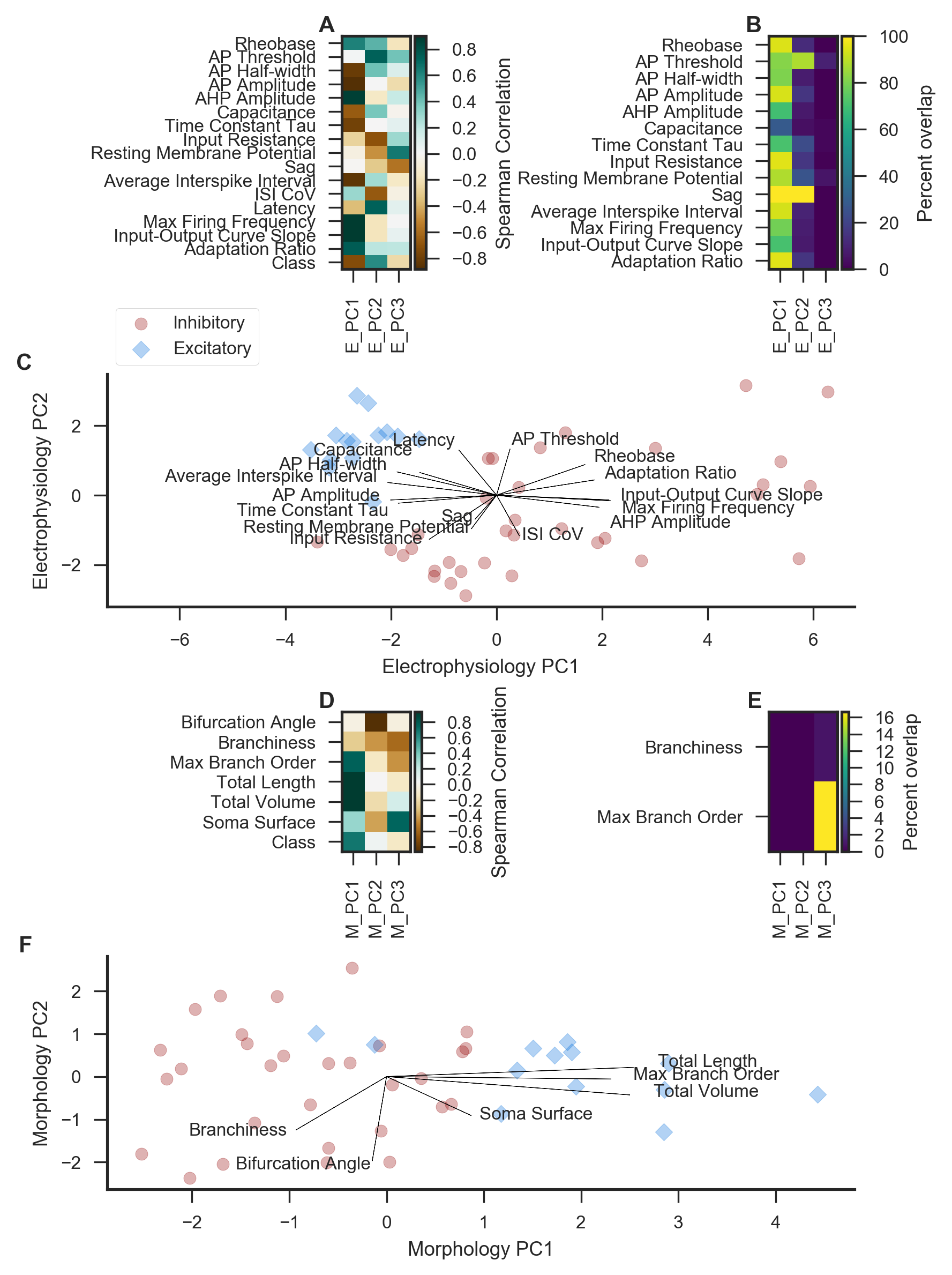

Supplement: S2 Fig — (A) Spearman correlations between the first 3 principal components (PC) calculated based on the set of electrophysiological properties (E_PC1-3) versus the properties themselves. (B) Percent overlap between each electrophysiological property with each PC. In other words, out of the genes which are significantly associated with the property at FDR = 0.1, the percentage which are also significantly associated with the indicated PC at FDR = 0.1. Properties for which there were no significant genes are not shown. (C) Vector map showing the strength of the association between each electrophysiological property and the first two PCs. Points represent individual cells types plotted according to their values of the first two PCs. (D-F) Same as A-C, but for morphological properties. (TIFF) [file pcbi.1007113.s002.tiff]

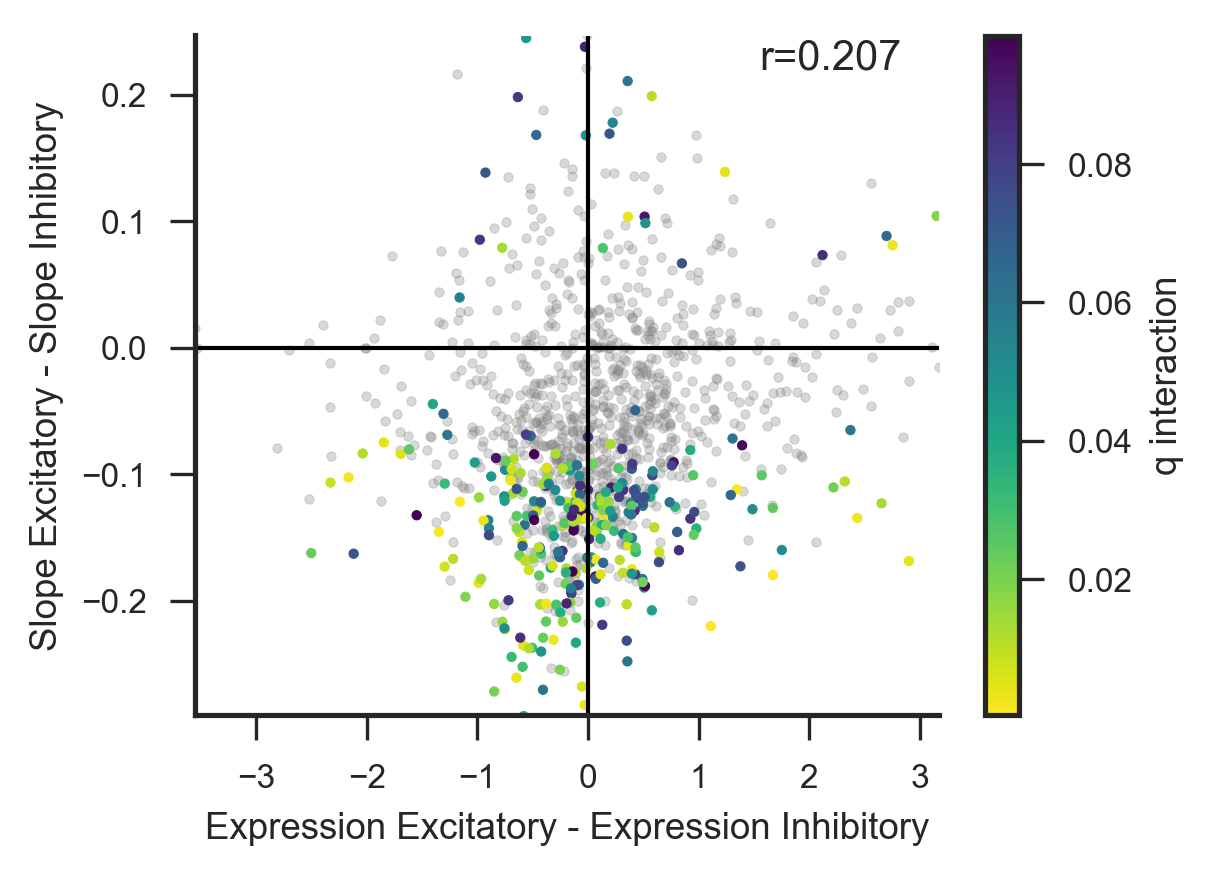

Supplement: S3 Fig — Between-class differences in gene expression plotted against differences in gene-property slope in the interaction model for the property AHP amplitude. Each point represents a single gene; grey points do not have a significant interaction and others are colored according to their significance level in the interaction model. For clarity of visualization only a random subset of the data (10% of the total number of genes) are plotted. (TIFF) [file pcbi.1007113.s003.tiff]
