# Supplementary material for: Transcriptomic correlates of electrophysiological and morphological diversity within and across excitatory and inhibitory neuron classes
Source: PLoS Comput Biol. 2019 Jun 18;15(6):e1007113. doi: 10.1371/journal.pcbi.1007113 (PMC6599125; doi:10.1371/journal.pcbi.1007113)
Supplement: S4 Table — “Muñoz-Manchado” refers to the dissociated cell dataset [15] which was used as a reference atlas to define the cell types in the PatchSeq dataset from the same work. The Allen Institute dataset [20] was used as the reference atlas for all other PatchSeq datasets, which were obtained from neocortical or hippocampal cell types. (DOCX) [file pcbi.1007113.s007.docx]

| Dataset | Subclass |
| --- | --- |
| Allen Institute (Neocortex) | Vip |
|  | Lamp5 |
|  | Sst |
|  | Sncg |
|  | Serpinf1 |
|  | Pvalb |
|  | Meis2 |
| Muñoz-Machado (Striatum) | CHAT |
|  | MSND1 |
|  | MSND2 |
|  | Mia |
|  | Pthlh |
|  | Sst |
|  | Th |
